# Pain management for a case with Nutcracker syndrome, undergoing a newly developed bypass surgery

**DOI:** 10.1186/s40981-021-00453-9

**Published:** 2021-06-14

**Authors:** Lieven Ameye, Joao Amaral, Armando Lorenzo, Kazuyoshi Aoyama

**Affiliations:** 1 Department of Anesthesia and Pain Medicine, The Hospital for Sick Children, Toronto, Canada; 2 Department of Diagnostic Imaging – Division of Interventional Radiology, The Hospital for Sick Children, Toronto, Canada; 3 Department of Urology, The Hospital for Sick Children, Toronto, Canada; 4 Child Health Evaluative Sciences, Peter Gilgan Centre for Research and Learning, The Hospital for Sick Children Research Institute, Toronto, Canada

**To the Editor**

Nutcracker syndrome (NCS) is a rare condition characterized by a group of clinical manifestations, like hematuria and pain, and is most commonly due to compression of a left renal vein (LRV) between the superior mesenteric artery and the aorta. NCS is an important differential diagnosis of abdominal pain in young females because of its relatively high prevalence [1, 2]. Chronic pain management is pivotal from the diagnosis of NCS to the end of treatment because the overall clinical course generally takes several years [1, 2]. We herein present a case of NCS, who underwent a newly developed bypass surgery, describing the transition of its pain management.

A 17-year-old female presented with a 3-month history of difficulty urinating, daily painless gross hematuria, loin and back pain, nausea and vomiting, and weight loss. She described the pain as diffuse abdominal, present at all times, without any exacerbating or mitigating factors. Based on the history and clinical presentation, she required imaging to diagnose NCS.

Her Doppler ultrasonography and MRI showed the LRV stenosis consistent with NCS (Fig. 1A). A venogram under general anesthesia (GA) confirmed the diagnosis and demonstrated collateral drainage for possible bypass surgery. This study confirmed an area of compression of the LRV before entering the inferior vena cava: this was demonstrated as a lower contrast density area (“white area”) in the LRV due to the compressed vein and smaller space for contrast to flow through (Fig. 1B). Perinephric collateral veins abnormally developed, likely due to partial obstruction and flow resistance. Because of her refractory pain despite chronic pain management over 7 months, including PO acetaminophen 1000 mg and PO morphine 10 mg both as needed, she elected to undergo a newly developed bypass surgery of her gonadal vein into the ipsilateral external iliac vein [3].

**Fig. 1:**
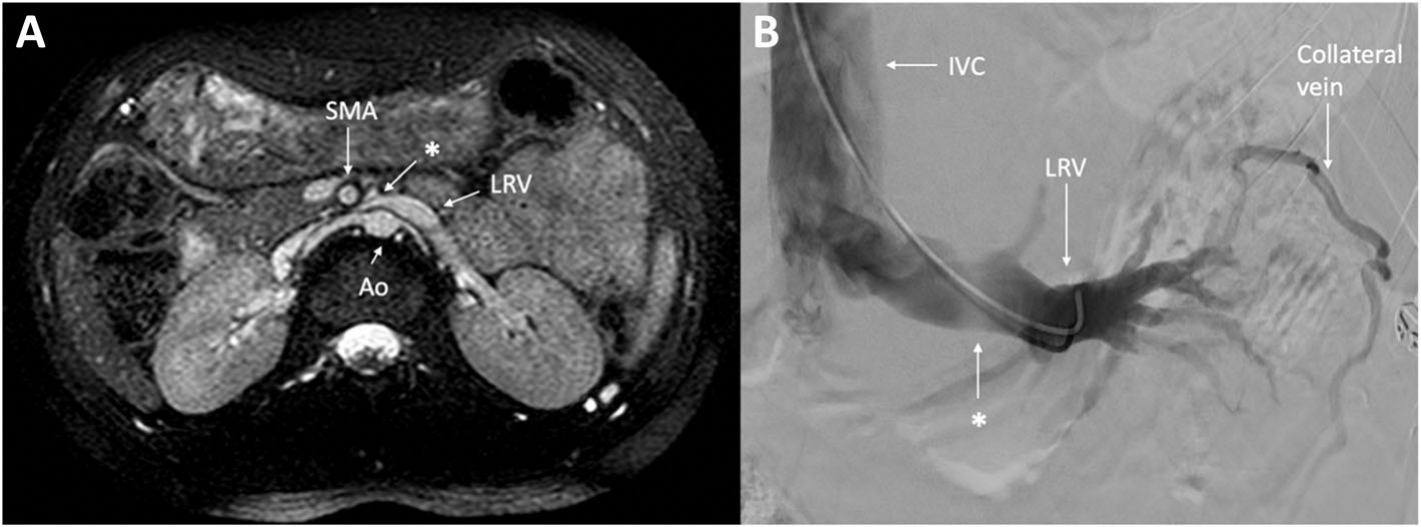
**A** Magnetic resonance imaging of the kidneys and renal veins. Note the narrowing/pinching (*) between the SMA and the anterior aorta in the left renal vein (LRV) with a dilation proximal to the narrowing. **B** Venogram of the left renal vein. Note the remarkable low contrast density (*) before entry into the IVC, due to compression by the SMA and the anterior aorta. The perinephric vein of collateral drainage, due to partial obstruction and flow resistance, is abnormally observed. Ao, aorta; SMA, superior mesenteric artery; IVC, inferior vena cava; LRV, left renal vein

The team of urology, cardiovascular surgery, and radiology carried out her bypass surgery. She was 171 cm and 60 kg on the day of the surgery. GA was induced with propofol 150 mg and fentanyl 100 μg, followed by tracheal intubation and was maintained with sevoflurane. Left-side transverse abdominis plane block was performed with 0.25% bupivacaine 20 ml with adrenaline and dexmedetomidine 30 μg for intraoperative pain management, in addition to intravenous morphine 2 mg. GA for the bypass was uneventful and required 7 h and 40 min in total. Her pain had improved (visual analog scale: 5 to 6) with less pain management, including PO acetaminophen 1000 mg and PO morphine 5 mg both as needed, on postoperative day 30. PO morphine was discontinued 90 days after the surgery.

This case was the second successful case of newly developed bypass surgery. In the first case, the pain had entirely resolved after bypass surgery [3]. Hence, bypass surgery can be a new surgical pathway for patients with refractory pain due to NCS, as bypass surgery is less invasive than a definitive surgical procedure such as an auto renal transplant.

## Data Availability

All available data and materials are presented in the main document.
